# Local Treatment of Diphtheria

**Published:** 1893-12

**Authors:** 


### LOCAL TREATMENT OF DIPHTHERIA.

SIMON (*L'Union Medicale*, June 3, 1893) has employed the following application with considerable success in the treatment of diphtheria:

| | | |
|---|---|---|
| ℞. | Salicylic acid, | gr. xv; |
| | Infusion of eucalyptus, | |
| | Glycerin, of each, | ℥ iss; |
| | Alcohol, enough to make a solution. | |

After applying this thoroughly to the affected parts he subsequently paints them with a solution of perchloride of iron and glycerin, in equal parts. He also employs in conjunction with this treatment irrigation of the nose and mouth, using a solution of boric acid or a 1 per cent. aqueous solution of carbolic acid, He also believes it advantageous to vaporize a decoction of eucalyptus leaves or a solution of thymol and water. For fissures and cracks of the lips and gums and for the inflammation following the removal of the pseudo-membrane, he uses the stick of silver nitrate to effect rapid healing. If cutaneous inflammations follow diphtheritic inflammation, he uses tincture of iodine or an alkaline solution of iodoform.—*Ex.*

